# Fretting Fatigue Experiment and Analysis of AlSi9Cu2Mg Alloy

**DOI:** 10.3390/ma9120984

**Published:** 2016-12-05

**Authors:** Jun Wang, Hong Xu, Tiexiong Su, Yi Zhang, Zhen Guo, Huping Mao, Yangang Zhang

**Affiliations:** 1 School of Mechanical and Power Engineering, North University of China, Taiyuan 030051, China; sutiexiong@nuc.edu.cn (T.S.); zhangyi_taiyuan@163.com (Y.Z.); maohp@nuc.edu.cn (H.M.); zyg31124@163.com (Y.Z.); 2 School of Materials Science and Engineering, North University of China, Taiyuan 030051, China; xh725@263.net; 3 China North Engine Research Institute, Tianjin 300400, China; guozhen@163.com

**Keywords:** fretting fatigue, fractography, fretting scar, fretting debris, micro-hardness

## Abstract

An investigation was carried out in order to study the fretting fatigue behavior of an AlSi9Cu2Mg aluminum alloy. The fretting fatigue tests of AlSi9Cu2Mg were performed using a specially designed testing machine. The failure mechanism of fretting fatigue was explored by studying the fracture surfaces, fretting scars, fretting debris, and micro-hardness of fretting fatigue specimens using scanning electron microscopy (SEM), energy-dispersive X-ray spectroscopy (EDX), and micro Vickers hardness test techniques. The experimental results show that the fretting fatigue limit (42 MPa) is significantly reduced to approximately 47% of the plain fatigue limit (89 MPa) under 62.5 MPa contact pressure. Furthermore, the fretting fatigue life decreases with increasing alternating stress and increasing contact pressure. The examination results suggest that the stress concentrates induced by oxidation-assisted wear on the contact interface led to the earlier initiation and propagation of crack under the fretting condition.

## 1. Introduction

Fretting is induced by vibrations or the application of bulk fatigue stresses to one or both of the contacting parts, and widely occurs at the contact interface between two parts when there is small amplitude oscillatory relative movement (typically 5–50 micrometers), such as dovetail roots of compressor blades in aircraft engines [1,2,3,4], cables in overhead conductors [5] and mine hoists [6], riveted joints [7], prostheses [8], etc. Many studies have shown that the combined actions of fretting and fatigue had a devastating effect on the fatigue limit of metallic materials, including aluminum alloys [7]. Fretting results in an enormous reduction of the endurance limit of a component by half or more, in comparison to the normal fatigue conditions [9]. In general, the main parameters governing fretting and fretting fatigue damage are known as cyclic axial loading, contact pressure, relative slip motion, and surface conditions, etc. Considerable research attention has been given towards developing an understanding of the influences of various parameters on fretting fatigue strength. Ramakrishna Naidu et al. [10] studied the effects of contact pressure on the fretting fatigue life of Al-Mg-Si alloy. With an increase in contact pressure, fretting fatigue life decreased, showed a minimum at 100 MPa contact pressure, then increased to a maximum at an intermediate contact pressure of 150 MPa, and thereafter decreased. The author attributed this variable behavior to the change in frictional stress, critical relative slip, crack growth retardation effect due to crack closure at high contact pressures, and stress concentration. Iyer [11] studied the relative effects of peak contact pressure, cyclic stress amplitudes, contact semi-width, and slip amplitude on fretting fatigue life of Ti-6Al-4V based on cylinder-on-flat contact configuration by means of a finite element model and fretting fatigue experiments. Muthu [12] observed that the crack initiation life and propagation life of 7075-T6 aluminium alloy decreases with an increase in cyclic axial stress. Majzoobi [1] studied the improvement of fretting fatigue resistance of AL-7075 using surface treatments, including nitriding and titanium coating. Vázquez et al. [13,14] studied the effect of peening (shot and laser) and textured surface on fretting fatigue in the Al 7075-T651 alloy. The results show the beneficial effect of the compressive residual stresses induced by surface treatments and the improvement that surface roughness or texturisation causes in fretting fatigue life.

Vehicle engines tend to have a design of lightness and high specific volume power, and the component parts work under increasingly harsher conditions [15]. In place of cast iron, aluminum alloy is used as the material of engine cylinder blocks to meet the continuous demands for better fuel efficiency, lower thermal-expansion coefficient, and cleaner exhaust, since the cylinder blocks are the largest and most intricate single piece of metal used in an internal combustion engine (ICE), on which other important engine parts are mounted. As a result, fretting wear occurs frequently on the aluminous cylinder block, where engine vibration causes a relative slip between the contact surfaces of blocks and main bearing caps, which can accelerate fretting and fatigue failures by creating crack initiation sites [16,17,18]. AlSi9Cu2Mg aluminum alloy is widely used for vehicle engine blocks due to its excellent properties, such as high heat resistance, good toughness, and excellent casting and machining features [19,20]. It is necessary to study the fretting fatigue performance of AlSi9Cu2Mg, but unfortunately there are few reports on its fretting fatigue so far.

The present investigation was undertaken to study the fretting fatigue performance and mechanisms of AlSi9Cu2Mg aluminum alloy. The research background of the project was the fretting fatigue damage in engine aluminum blocks, and the aim was to provide accurate data to support theoretical design and the practical application of AlSi9Cu2Mg alloy in vehicle engines. The main research was conducted in the following aspects. A new fixture was designed and manufactured typically for the fretting fatigue test, which has enough accuracy and reliability and enabled testing conditions closer to actual working condition of the engine block. The fretting and plain fatigue behaviors were evaluated to reveal the effect of fretting condition on the fatigue performance of AlSi9Cu2Mg alloy. Fatigue fracture, fretting scar, fretting debris, and micro-hardness of fretting fatigue specimens were analyzed by SEM, energy-dispersive X-ray spectroscopy (EDX), and micro Vickers hardness test techniques to characterize the mechanisms of fretting fatigue of AlSi9Cu2Mg alloy.

## 2. Experimental Fixture and Experiments

### 2.1. Experimental Fixture

To investigate the characteristics of fretting fatigue, various test apparatus have been developed for special purposes in different fields. Literature on fretting fatigue clearly demonstrate that the fatigue load is imposed mostly by the hydraulic actuator [3], and occasionally by the position control machine [5,6,9]; the contact pressure of fretting specimens is induced through the clamping pads attached to the proving ring [1,21,22], spring [23], or other fretting fixtures [3,9,12,24,25]. Geometries of the pads are flat, spherical, cylindrical [25], and bridge-type [5,6,21,26], but most specimens have a flat fretting contact surface. Though there are diverse apparatus for fretting fatigue tests, the design has to meet certain requirements to obtain efficient operation and results [24]: (i) the ability to produce a constant normal force between specimens and pads, and the visibility of pad and specimen; (ii) a few microns relative movements; (iii) very high stiffness; (iv) high precision of measurement.

**Figure 1:**
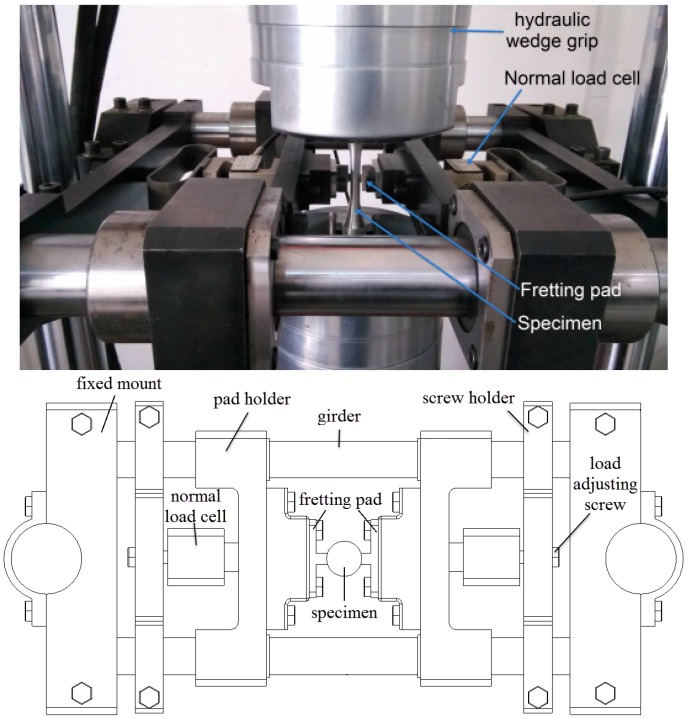
A schematic of the fretting fatigue test fixture.

A fretting fatigue test fixture has been developed by the authors in this investigation. As shown in Figure 1, the schematic of the fixture mainly consists of two fixed mounts, two girders, two normal load cells, two load adjusting screws, two screw holders, two fretting pad holders, and two fretting pads, etc. The fixed mounts and girders constitute the supporting platform of the fretting fatigue fixture, which is completely tightened on the two main columns of the HYS-100 servo-hydraulic testing machine (Changchun Haoyuan Industry Co. Ltd., Changchun, China) by the screws. The other parts of the fixture are installed on the platform.

The fretting pads are fastened on the pad holders, which can always move along the girders. The adjusting screw holders can also move along the girders, to facilitate the installation of the specimen before the test. When a specimen is installed by the hydraulic wedge grip on the fatigue testing machine, the screw holders and the pad holders slide just to make the pad against the flat specimen. Then, the screw holders are fixed on the girders. The normal loads—which are measured in real-time using two load cells—are induced by two adjusting screws, which impel the fretting pad to clamp the specimen tightly. The load cell digital readings can ensure that the contact loads in two opposite directions are equal and sufficiently accurate.

**Figure 2:**
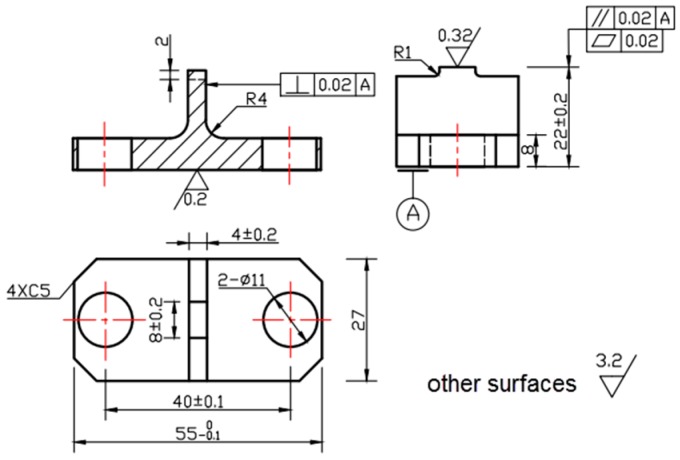
Dimensioned design drawings for the fretting pad which was used to transmit the contact pressure; dimensions are in mm.

Figure 2 shows the geometry of the fretting pad, including the details of machining and surface condition. The contact surfaces of the pad are rectangular with width 4 mm and length 8 mm, and the surface roughness is less than 0.32 µm, which was obtained by low stress grind machining and polishing processes. Any possible effect from surface defects during the machining was eliminated.

To make the laboratory conditions closer to actual working conditions, the parallelism between the contact surfaces of the specimen and pad was maintained, and complete flat-on-flat contact was achieved. The fretting fatigue fixture has the following characteristics: (i) it can simulate flat-flat or round-flat contact states between specimens and pads by using different types of pads with plane or cylindrical geometries; (ii) it produces a constant normal force of maximum 5 kN between pads and specimens; (iii) it has visibility of the pads and the specimens; (iv) its normal load can be measured using compression load cells with the accuracy of ±1% FSO (full-scale output), which corresponds to ±0.05 kN.

### 2.2. Materials

AlSi9Cu2Mg aluminum alloy specimens machined from casting die round bars (diameter 27 mm, length 170 mm) after heat treatment (referred to as T6 condition, solid solution treatment and complete artificial aging were done at temperatures of 520 °C and 175 °C for 12 h and 7 h, respectively) were used in fretting and plain fatigue tests. The composition of AlSi9Cu2Mg is shown in Table 1. Commercial 42CrMo high strength structural steel was used as the material for the fretting pads. The composition of 42CrMo is shown in Table 2. The mechanical properties of AlSi9Cu2Mg and 42CrMo are given in Table 3. One aim of our research was to simulate the practical case of engine block and main bearing cap, where the material of the block is AlSi9Cu2Mg and the main bearing cap is 42CrMo. The choice of the materials for the fretting specimen and the pad was made to match this real application case.

**Figure 3:**
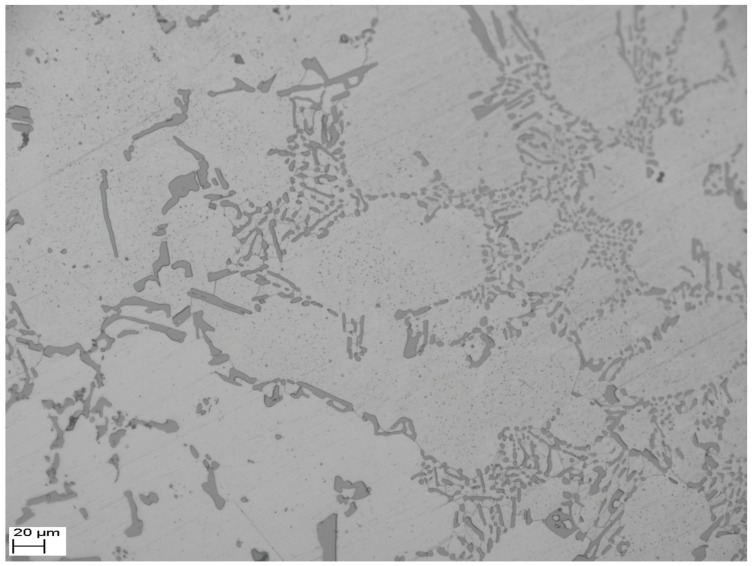
A SEM microstructure graph of the AlSi9Cu2Mg alloy.

The AlSi9Cu2Mg aluminum alloy used in this application has the microstructure shown in Figure 3. The metallographic samples cut from the fretting specimens were prepared by coarse grinding, accurate grinding, and mechanical polishing. Etching was carried out using 0.5% hydrofluoric acid aqueous solution for 7 s. Then, the samples were rinsed with anhydrous alcohol and drying. The micrograph was obtained using a Zeiss Axiovert 200 MAT metallographic microscope (Zeiss Group, Oberkochen, Germany). The lighter substrate is aluminum limited solid solution (∝ phase), and the reticular structures are metal compounds (strengthening phase).

**Figure 4:**
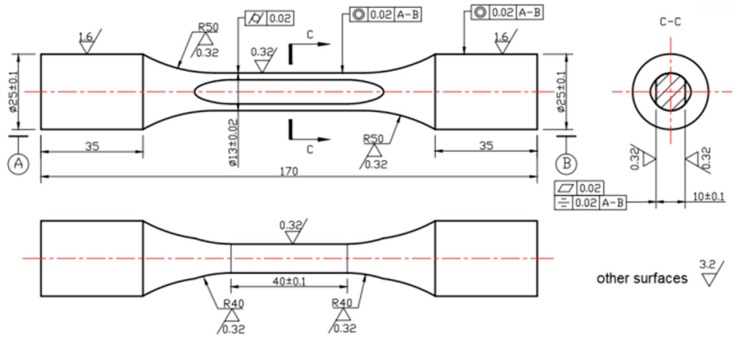
Dimensioned design drawings for the fretting specimen; dimensions are in mm.

Figure 4 shows the geometry of the fretting fatigue specimen, including the details of machining and surface condition. The specimen has a gauge length of 40 mm, a width of 13 mm, and a thickness of 10 mm. The gauge portions of all specimens were polished with SiC papers and cleaned with acetone. The average roughness of the specimen contact surface in the transverse direction is 0.32 µm. Such a procedure allows the elimination of the remaining circumferential notches that could act as stress concentrators during plain and fretting fatigue tests.

### 2.3. Experiments

In this investigation, all the cyclic loads of the plain and fretting fatigue tests were set as constant amplitude sinusoidal loads with a frequency of 10 Hz and a stress ratio of *R* = 0.1 in the HYS-100 fatigue testing machine (Changchun Haoyuan Industry Co. Ltd., Changchun, China). All experiments were carried out to specimen failure or 10^7^ cycles in atmospheric conditions at room temperature. The number of cycles was recorded. Specifically, for fretting fatigue tests, the fretting pad pressed against the specimen to simulate fretting condition.

The contact pressure (σ_n_) was maintained at 62.5 MPa (equal to the pressure between the engine block and the main bearing cap at the engine installation condition) in flat-flat contact state, which was equivalent to clamping forces (*F_c_*) of 2 kN and applied by the adjusting screws. Nine different maximum alternating stress loads (σ_max_) at 51.88, 60.52, 69.17, 77.82, 86.47, 103.75, 121.04, 138.34, and 155.63 MPa were tested, which were equivalent to maximum axial forces (*F*_max_) of 6, 7, 8, 9, 10, 12, 14, 16, and 18 kN, respectively.

Considering the scatter of experimental lives, the group method was used for fatigue tests at each stress level [15]. The median fatigue life (*N*_50_) was calculated. The minimum specimen number (*n*) should satisfy the following rule:

(1) $$C_{V}<\delta \sqrt{n}/t_{a}$$

Further:

(2) $$C_{V}=S_{x}/\bar{x}$$

(3) $$S_{x}=\sqrt{\left(\sum_{i=1}^{n}x_{i}^{2}-\frac{1}{n}{\left(\sum_{i=1}^{n}x_{i}\right)}^{2}/\left(n-1\right)\right)}$$

(4) $$N_{50}=10^{\bar{x}}$$

where *C_V_* is the variation coefficient, δ is the margin of error (generally 5%), *t_a_* is the critical value of t-distribution, *x_i_* is the logarithm of the fatigue life of the *i*-th specimen, $\bar{x}$ is the average value of the logarithm of the fatigue life under a given stress level, and *S_x_* is the standard deviation. The *t_a_* is determined by the degree of freedom, *V* = *n* − 1, and the confidence level γ. The γ is also the probability value that the relative error of $\bar{x}$ is lower than *δ*.

The experimental plain and fretting fatigue limits were defined as the runout stress amplitude at which failure had not occurred at 10^7^ cycles. The staircase test method was employed. The step size of the stress amplitude was set to 3 MPa.

Furthermore, in order to study the influence of *F_c_* on the fretting fatigue life of AlSi9Cu2Mg, the fretting fatigue experiments were conducted at four different σ_n_ of 62.5, 93.75, 125, and 140.63 MPa, which was equivalent to *F_c_* of 2, 3, 4, and 4.5 kN. Simultaneously, σ_max_ was maintained at 69.17 MPa, which corresponded to *F*_max_ = 8 kN.

The observations of the fracture surfaces of plain and fretting fatigue specimens, the fretting region, and the fretting debris were carried out using a ZEISS EVO MA 15 SEM (Zeiss Group, Oberkochen, Germany). The debris was identified by Bruker EDS QUANTAX 200 (Bruker Corporation, Karlsruhe, Germany) energy-dispersive X-ray spectroscopy.

Micro-hardness on the fracture surface and fretting regions of fatigue specimens was measured using a digital electronic micro Vickers hardness tester (Shanghai Wanheng Precision Instruments Co. Ltd., Shanghai, China) with a diamond pyramid Vickers indenter. The test load was 50 g, and indenter dwell time was 15 s. The purpose was to examine the influence of fatigue and fretting on micro-hardness of different regions.

## 3. Results and Discussion

### 3.1. Fretting Fatigue Life

**Figure 5:**
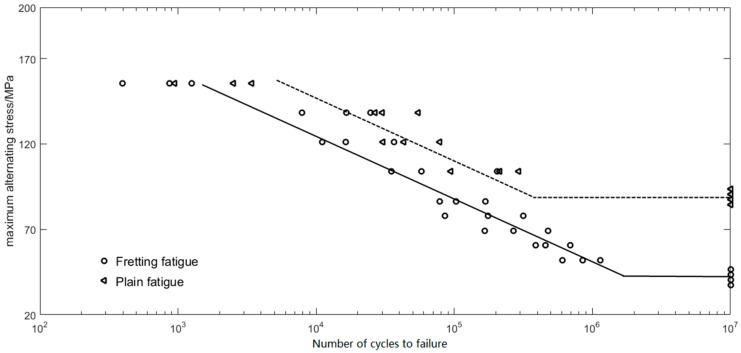
The S-N curves obtained from the experiments on AlSi9Cu2Mg aluminum alloy.

Figure 5 shows the S-N curves obtained from the experiments on AlSi9Cu2Mg aluminum alloy, which indicates that the plain and fretting fatigue lives both decrease with increasing alternating stress, the plain fatigue limit is 89 MPa, and the fretting fatigue limit is 42 MPa (which is only 47% of the plain fatigue limit), under the condition of σ_n_ = 62.5 MPa. The experimental plain and fretting fatigue lives with the confidence level 50% are listed in Table 4. Under the same alternating stress, the fretting fatigue life (*N*_f50_) is much lower than the plain fatigue life (*N*_f50_), and the percent decrease of fatigue life due to fretting is found to be 60% on average, and up to 62% under the alternating stress σ_max_ = 155.63 MPa. Furthermore, Table 5 shows the influence of normal stress σ_n_ on the fretting fatigue life *N*_f50_ under σ_max_ = 69.17 MPa. It proves that there is a significant impact of different contact pressures on lifetime when contact pressure increases, and *N*_f50_ is decreased sharply.

### 3.2. Fractographic Observations

**Figure 6:**
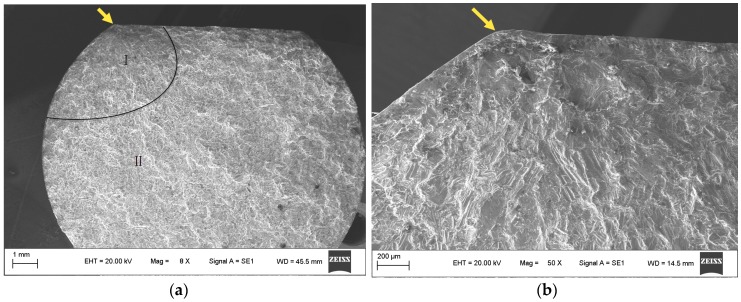

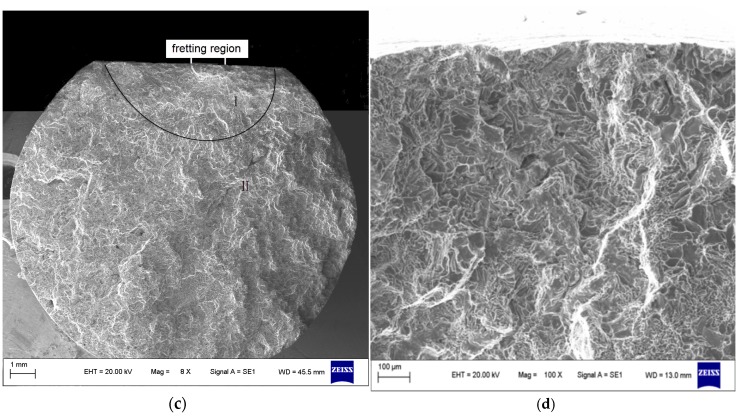
SEM micrographs of the fracture of AlSi9Cu2Mg. (**a**,**b**) Plain fatigue test specimen (*N_p_* = 8.4 × 10^5^); and (**c**,**d**) fretting fatigue test specimen (*N_f_* = 2.8 × 10^5^, σ_n_ = 62.5 MPa).

The typical fracture morphologies of the plain and fretting fatigue specimens are illustrated in Figure 6. The figure clearly indicates that the fracture surface consists of two quite distinct regions: a fatigue zone created by crack propagation and an instant rupture region which gives rise to fracture of specimens when it is sufficiently weakened by the crack zone development.

Figure 6a,b show that the plain fatigue crack initiated at the intersection of the plane and the cylinder of the specimens (shown by the arrow). The fatigue crack shows a fan-shaped radial propagation. Due to the low propagation rate of crack at the propagation zone, the fatigue crack was subjected to repeated extrusion and friction, where the fracture is more flat and smaller than the instant rupture region.

Microscopic observations of the fracture surfaces of the fretting fatigue specimens show that cracks originated from the contact region, expanded inward (exactly beneath the contact region), and then radially propagated (Figure 6c,d). The crack initiation area of fretting fatigue is larger in contrast to that of plain fatigue, which is a point in the intersection of two fabricated faces. A similar phenomenon was observed in other studies. Sadeler et al. [26] found that during fretting, cracks inevitably start from the same location at points adjacent to the edge of the fretting regions, while during plain fatigue, cracks originate randomly at one or several points around the periphery of the specimen case. This is because there is a stress raiser (i.e., a steep gradient, which can be seen as a singular point) at the edge of the contact for a flat-flat contact in fretting condition, which results in early nucleation of cracks at the pad tip. The stress distributions of the contact surface under fretting conditions have been mathematically obtained in the studies by Vázquez et al. [27]. The normal load induces a compressive stress, which restrains the crack initiation in the direction of the axial load. Simultaneously, the axial tensile stress, which enforces the crack initiation, was induced near the outer edge of the contact region. The interaction of the compressive and tensile stress at the inner and outer sides of the contact edge makes the steep stress gradient.

### 3.3. Fretting Scar and Debris

**Figure 7:**
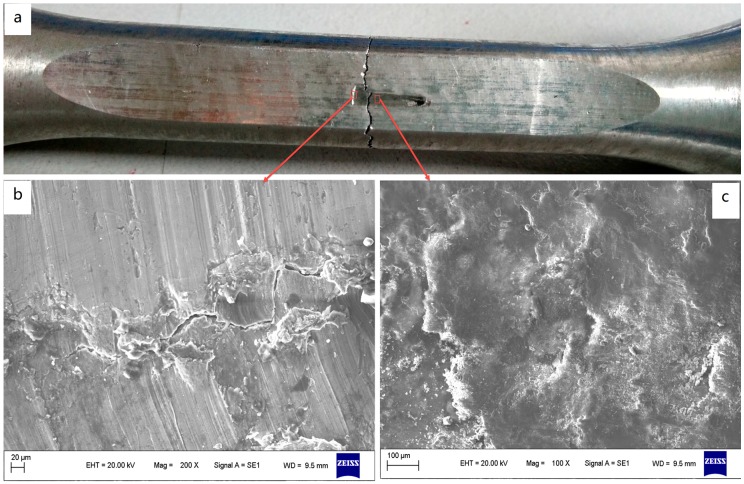
Fretting scar of fretting fatigue specimen. (**a**) Fretting scar and fracture position; (**b**) Morphology of scar, near the edge of fretting region; (**c**) Morphology of scar, far away from the edge of fretting region.

Fretting region morphology of the specimen is shown in Figure 7. The contact surface is worn black by fretting, and the fracture occurs at about 2 mm to the edge of the worn area (Figure 7a). As we can see, although the square punch pad and fretting fatigue specimen were designed for a flat-flat contact, full contact between them is hard to attain, and only a part of the region contacted in actual laboratory conditions. The flatness of the contacting surfaces was quantified by the surface roughness of the pad and specimen. So, the actual contact stress is larger than the nominal stress between pad and specimen, which increases the damage to the specimen in fretting conditions.

**Figure 8:**
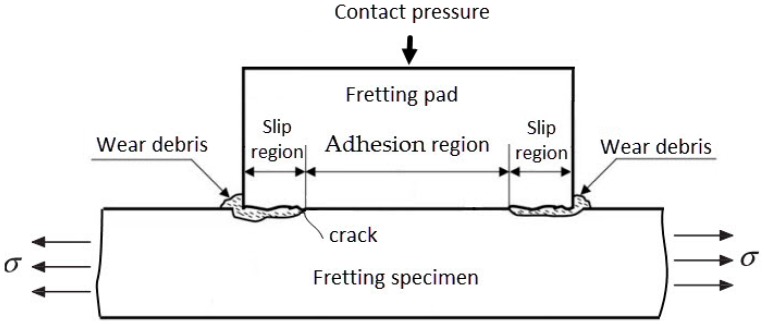
Schematic view of the contact region.

Investigations have shown that there are generally three regions on the entire contact interface, corresponding to an inner adhesion region and two slip regions under fretting condition [27], and the fretting cracks initiate predominantly at the boundary between slip and adhesion regions [28]. A schematic view of the fretting contact region is shown in Figure 8. Figure 7b,c show different morphologies of the fretting scars in different places of the fretting region: strip scratch and metal accumulation on the near edge of the contact region, and lamellar structure and plenty of small granular debris in the middle of the contact region. The strip scratch region corresponds to the slip region where the remarkable abrasive wear occurs, and the wear debris is swept out from the edge of the pad to pile up on the specimen surface. The lamellar structure region corresponds to the adhesion region of the contact interface. These trends are almost similar to the above references. Furthermore, the crack is observed on the edge of the contact region which was decided by the contact stress fields (details in Section 3.2). However, this crack is not the cause of specimen fracture in the fretting tests. The cause may be that since the contact pressure acts through uneven wear damaged parts, the total contact pressure acting in the slip region is lower than the average contact pressure of the pad. As a result, the total contact pressure acting in the adhesion region is higher than that in the slip region. Therefore, the stress concentration is considered to occur at the transition between slip and adhesion regions [29], which is the main cause of the specimen fracture.

**Figure 9:**
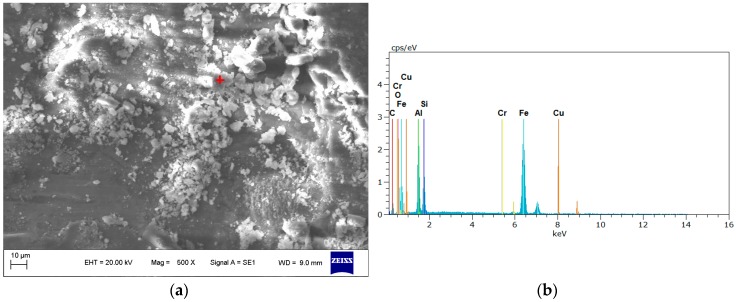
An example of EDX analysis of fretting debris. (**a**) SEM image indicating the area of analysis; (**b**) spectrum of the fretting debris.

The chemical composition of the fretting debris was further investigated using EDX apparatus, as shown in Figure 9. According to the results of the spectrum analysis, the debris was comprised of Fe 53.36%, Al 39.78%, Si 3.85%, O 1.87%, Cu 0.73%, and Mg 0.41% (in wt %). Fe element, which was detected on the fretting scar, was transferred from the 42CrMo pad. So, it is obvious that the debris is a mixture of both AlSi9Cu2MgA aluminum alloy and 42CrMo structure steel. Oxygen found in the wear debris was thought to come from the oxidation of the aluminum alloy which was caused by a huge amount of heat out of the fretting and friction between the specimen and pad in the larger contact stress condition. Fu et al. [30] have reported that during fretting, a huge amount of hard oxide debris accumulates on the contact surface and can cause severe abrasion. Tao et al. [31] have reported that the more serious the fretting is, the higher the oxidation degree of fretting debris.

As a result, although the stress raises at the edge of the contact and the stress concentrates on the fretting region, both can cause crack initiation and propagation; the oxidation-assisted wear mechanisms are predominant in the AlSi9Cu2Mg alloy specimen under the fretting condition. To be specific, with the alternating stress load on specimens, the particle-type debris ruptured from the lamellar structure of specimen and overflowed due to vibration and friction, eventually pitting the fretting contact area on the specimen. The pit brought severe stress concentration on the fretting fatigue specimen, which intensified the crack initiation and propagation.

### 3.4. Micro-Hardness

**Figure 10:**
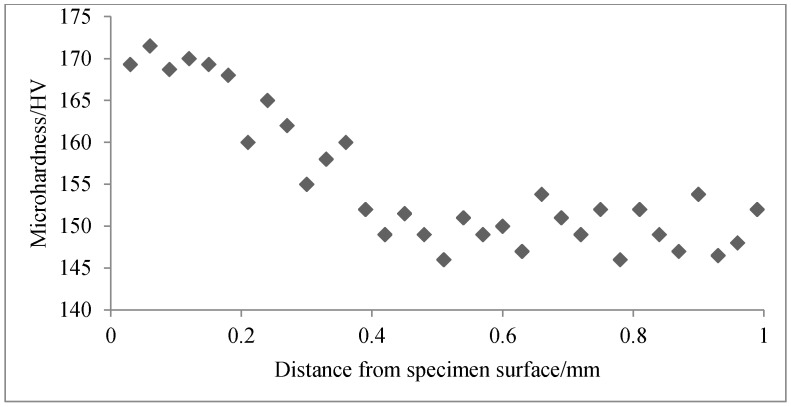
Micro-hardness depth profile of fretting fatigue fractured AlSi9Cu2MgA (*N_f_* = 2.8 × 10^5^, σ_n_ = 62.5 MPa).

The average micro-harnesses of the fretting region and the region away from fretting were 173 HV and 116 HV, respectively, from multi-point measurements on gauge plane of the specimens at $\sigma_{n}$ = 62.5 MPa. Figure 10 shows that the average micro-hardness from five measurements varies with the distance away from the specimen surface under the fretting region. Micro-hardness measurements were conducted on a polished fracture section with polishing cloth. It can be seen that the micro-hardness decreases from the surface underneath the fretting region—by about 13% from the first data point nearest to the surface to the stable alloy hardness (about 149 HV) approximately 0.5 mm under the surface of the fatigue specimen. This means that fretting affects the material up to 0.5 mm below the fretting surface.

The micro-hardness is higher than the specimen interior underneath the fretting damage surface, likely for the reasons discussed below. During fretting fatigue, the continuous larger contact pressure (62.5 MPa) and high frequency fretting between the fretting pad and the specimen generate small plastic deformation in the fretting contact region of the fatigue specimen. The small plastic deformation which repeated accumulation for a long time, leading to cold hardening and compaction of the near-surface metallic structure, thereby causing an increase in hardness. Otherwise, the micro-hardness is lower than the specimen interior in places far from the fretting damage region, probably because of the fatigue hardening effect caused by the cyclic load [31]. This hardening effect is stronger at the interior of the fatigue specimen.

## 4. Conclusions

(1). The fretting fatigue limit of AlSi9Cu2Mg alloy was 42 MPa under 62.5 MPa contact stress, which is 47% of the plain fatigue limit (89 MPa). The fretting fatigue crack initiated near the contact edge of the specimen.
(2). The fretting fatigue life of AlSi9Cu2Mg alloy decreases with increasing alternating stress. Simultaneously, the percent decrease of fatigue life due to fretting is up to 62% under the same alternating stress level.
(3). The oxidation-assisted wear mechanism is predominant in the AlSi9Cu2Mg alloy specimen under the fretting condition. With the alternating stress load on specimens, the combination effect of the stress raiser at the edge of the contact and the stress concentration on the fretting region brings the initiation and propagation of cracks. As a result, the fatigue strength and fatigue life of AlSi9Cu2Mg alloy sharply decline.
(4). The average micro-harnesses of the fretting region and the region away from the fretting are 173 HV and 116 HV, respectively. The micro-hardness is higher than the specimen interior underneath the fretting damage surface. The micro-hardness is lower than the specimen interior in places far from the fretting damage region.

## Figures and Tables

**Table 1:** Composition of AlSi9Cu2Mg aluminum alloy used for specimens.

Element	Si	Cu	Mg	Mn	Ti	Al
wt %	6–8	1.3–1.8	0.3–0.5	0.1–0.3	0.001–0.0025	Balance

**Table 2:** Composition of 42CrMo high strength structural steel used for pads.

Element	C	Mn	Si	Cr	Mo	P	S	Fe
wt %	0.38–0.45	0.50–0.80	0.17–0.37	0.90–1.20	0.15–0.25	≤0.035	≤0.035	Balance

**Table 3:** Mechanical properties of AlSi9Cu2Mg and 42CrMo.

Materials	Young’s Modulus, GPa	Yield Strength, MPa	Ultimate Strength, MPa	Poisson’s Ratio	Elongation,%
AlSi9Cu2Mg	69	178	290	0.31	4
42CrMo	206	≥930	≥1080	0.28	≥12

**Table 4:** Lives obtained from the experiments on fretting and plain fatigue.

*F*_max_ (kN)	σ_max_ (MPa)	σ_n_ = 62.5 MPa			σ_n_ = 0 MPa			(1 − *N*_f50_/*N*_p50_)%
		*N_f_* (Cycles)	*N*_f50_ (Cycles)	*C_V_*	*N_p_* (Cycles)	*N*_p50_ (Cycles)	*C_V_*	
6	51.88	1,135,515	837,007	0.023	>10^7^	-	-	-
		853,561						
		605,006						
7	60.52	692,825	496,635	0.023	>10^7^	-	-	-
		456,878						
		386,980						
8	69.17	476,179	277,014	0.042	>10^7^	-	-	-
		269,412						
		165,698						
9	77.82	315,701	168,030	0.055	>10^7^	-	-	-
		85,272						
		176,230						
10	86.47	168,030	110,765	0.033	>10^7^	-	-	-
		103,090						
		78,452						
12	103.75	34,954	74,452	0.081	94,117	180,588	0.048	59
		57,643			213,990			
		204,825			292,418			
14	121.04	11,061	18,792	0.062	43,293	46,945	0.044	60
		16,327			30,590			
		36,745			78,122			
16	138.34	7902	14,805	0.060	54,830	35,592	0.037	58
		24,825			26,641			
		16,542			30,246			
18	155.63	865	756	0.088	3423	2013	0.088	62
		398			946			
		1254			2519			

**Table 5:** The influence of normal stress σ_n_ on the fretting fatigue life *N*_f50_ (σ_max_ = 69.17 MPa, *F*_max_ = 8 kN).

*F_c_* (kN)	σ_n_ (MPa)	*N_f_* (Cycles)	*N*_f50_ (Cycles)	*C_V_*
2	62.5	443,579	477,014	0.024
		669,215		
		365,643		
3	93.75	435,712	280,018	0.033
		190,127		
		265,042		
4	125	249,244	156,074	0.037
		146,563		
		104,075		
4.5	140.63	120,421	85,326	0.033
		90,645		
		56,912		
